# Increasing workload in Norwegian general practice – a qualitative study

**DOI:** 10.1186/s12875-019-0952-5

**Published:** 2019-05-21

**Authors:** Ellen Rabben Svedahl, Kristine Pape, Marlen Toch-Marquardt, Lena Janita Skarshaug, Silje-Lill Kaspersen, Johan Håkon Bjørngaard, Bjarne Austad

**Affiliations:** 1 Department of Public Health and Nursing, Faculty of Medicine and Health Sciences, Norwegian University of Science and Technology, Håkon Jarls gate 11, 7030 Trondheim, Norway; 2 SINTEF Digital, Department of Health, Trondheim, Norway; 3 Forensic Department and Research Centre Bröset, St. Olav’s University Hospital Trondheim, Trondheim, Norway

**Keywords:** General practitioner, GP, General practice, Family medicine, Workload, Coordination reform, Norway, Qualitative, Interview study

## Abstract

**Background:**

General practitioners (GPs) play a key role in securing and coordinating appropriate use of healthcare services, by providing primary and preventive healthcare and by acting as gatekeepers for secondary healthcare services. Historically, European GPs have reported high job satisfaction, attributed to high autonomy and good compatibility with family life. However, a trend of increasing workload in general practice has been seen in several European countries, including Norway, leading to recruitment problems and concerns about the well-being of both GPs and patients. This qualitative interview study with GPs and their co-workers aims to explore how they perceive and tackle their workload, and their experiences and reflections regarding explanations for and consequences of increased workload in Norwegian general practice.

**Methods:**

We conducted seven focus groups and four individual interviews with GPs and their co-workers in seven GPs’ offices in Mid-Norway: three in rural locations and four in urban locations. Our study population consisted of 21 female and 12 male participants; 23 were GPs and 10 were co-workers. The interviews were analysed using systematic text condensation.

**Results:**

The analysis identified three main themes: (1) Heavy and increasing workload – more trend than fluctuation?; (2) Explanations for high workload; (3) Consequences of high workload. Our findings show that both GPs and their co-workers experience heavy and increasing workload. The suggested explanations varied considerably among the GPs, but the most commonly cited reasons were legislative changes, increased bureaucracy related to documentation and management of a practice, and changes in patients’ expectations and help-seeking behaviour. Potential consequences were also perceived as varying, especially regarding consequences for patients and the healthcare system. The participants expressed concerns for the future, particularly in regards to GPs’ health and motivation, as well as the recruitment of new GPs.

**Conclusions:**

This study found heavy and increasing workload in general practice in Norway. The explanations appear to be multi-faceted and many are difficult to reverse. The GPs expressed worries that they will not be able to provide the population with the expected care and services in the future.

## Introduction

General practitioners (GPs) play a key role in securing and coordinating appropriate use of healthcare services, both by providing primary and preventive care and by acting as gatekeepers for secondary care services [1]. Previously, European GPs have reported high job satisfaction [2–7], largely attributed to high autonomy [8, 9] and good compatibility with family life [10]. However, a trend of increasing workload in general practice has been seen in several European countries [11, 12]. In England, studies report long and intense working hours, recruitment problems [13] and concerns for the well-being of both GPs and patients [14].

Several possible mechanisms explaining the increasing workload in general practice have been suggested [15]. In many European countries, healthcare reforms have transferred numerous tasks and responsibilities to primary care in order to reduce pressure on secondary care [16]. This implies that primary care now has increased responsibility for severely ill patients [17]. In addition, it has been suggested that new developments and treatment possibilities, as well as rising public expectations, have increased GPs’ workload [15].

The Regular GP scheme was introduced in Norway in 2001. This list-based system entitles all inhabitants to register with a regular GP, and it has been regarded as one of the most successful public services in Norway [18], with high satisfaction among both patients and GPs [19–21]. Most GPs are self-employed, and the reimbursement system is based on a combination of capitation fees and fee-for-service [22]. About 10% of GPs are employed by their local municipality and get a fixed salary [23]. The regular GPs are responsible for coordinating healthcare services for the patients on their lists, and medical attestation and follow-ups for all absence from work of 3–8 days, including attestation for absence from high school. In 2012, a Coordination Reform was implemented, delegating more tasks to general practice. On average, a GP’s patient list in Norway has approximately 1100 patients. This number has decreased in recent years [23], which may be a consequence of increased workload [24].

There is limited research on how increasing workload and the transfer of responsibilities to primary care may influence Norwegian general practice. This qualitative study aims to explore how GPs and their co-workers in Norway perceive and tackle their workload, and their experiences and reflections regarding explanations for and consequences of increased workload in general practice.

## Material and method

### Design

As this study is part of a project investigating different aspects of capacity pressure on health services [25], we wanted to identify possible mechanisms related to workload. We chose a qualitative method in order to explore and provide rich descriptions of these complex phenomena [26]. We applied a phenomenological approach, a methodology that relies on first-person accounts as the source of knowledge [27]. We collected data through interviews in urban and rural municipalities of Mid-Norway. We chose to conduct both focus groups and individual interviews for practical reasons, as not all of our participants in the same location could partake in interviews at the same time. In addition, we saw this as an opportunity to explore and compare dynamics when statements were given in groups as opposed to individual interviews.

### Participants

The study participants were recruited by strategic sampling, via personal invitations by e-mail. We aimed to include GPs with varying sex, age, experience, size of practice, managing style and geographical location, thus securing a wide range of perspectives on the topic. To enlighten the topic further, we also included co-workers (health secretaries and nurses) from some of the practices in the interviews. A total of 23 GPs and 10 co-workers were interviewed, and the focus groups consisted of participants working at the same office. For the characteristics, see Table 1. Each participant cited is referred to with an individual number, as well as denoting the number of the focus group (G) in which they were interviewed or if they were interviewed individually (I).

**Table 1:** Characteristics of Participants

	*N* = 33
Sex	
Female	21
Male	12
Occupation	
GP	23
Co-worker	10
Age	
20–29	6
30–39	7
40–49	12
50–59	3
60–69	2
missing	3
Location	
Rural	16
Urban	17
GP characteristics	*N* = 23
Years as a GP	
< 2	5
2–4	1
5–9	7
10–19	8
≥20	2
List size	
< 900	3
900–999	4
1000–1099	5
1100–1199	3
1200–1299	4
1300–1399	1
1400–1499	1
≥1500	1
No list/intern	1
Speciality	
General practice^a^	13
Other	1
No	9
Days per week in the office	
2–3	6
4–5	17

^a^Completed 5 years of speciality training in general practice, and mandatory courses

### Data collection

**Fig. 1:**
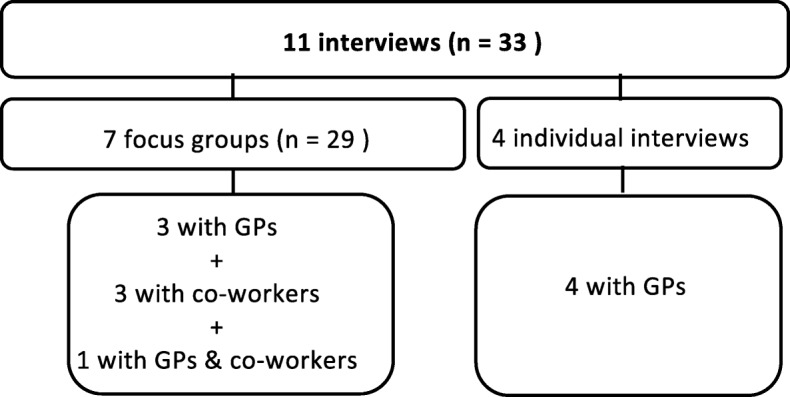
Data collection

We conducted 11 interviews in Mid-Norway between September 2017 and January 2018 (Fig. 1). The interviews were held at the practices and lasted approximately 60 min. The authors alternated as the main interviewers, and at least one medical doctor participated in each interview. We used a semi-structured interview guide (Table 2), pilot tested by an academic GP. The interview guide was adjusted continuously throughout the study. Further, we followed up on statements made by previous participants, exploring if other participants shared the same experience. Interviews were audio recorded and transcribed verbatim by a secretary. All audio records were listened to and transcripts were anonymised, as well as being proofread by at least one of the authors. Interviews were reviewed throughout the study, and they continued until we agreed that sufficient information power was reached and no new themes were emerging [28].

**Table 2:** Original interview guide

- How would you describe your GP office? o Compared to others? - How is your working situation right now? o How busy are you nowadays, in terms of workload? - Describe a regular day, compared to a particularly busy day at work. - Have you experienced situations that resulted in extreme time pressure? Which situations? - What might be consequences of time pressure / increased workload for you? - What kind of support do you get from your colleagues when you are out of time? - Imagine a **day** when you were under particular time pressure o How did you handle the situation? o How did you prioritise? - Imagine a **period** when you were under particular time pressure / had an increased workload. o How did you handle the situation? o How did you prioritise? - Which patient groups take up most of your time or take a lot of time to treat? - Considering your situation today, to what degree does time pressure / workload affect you? - Do you have any suggestions on how to reduce the workload for GPs? - What is your experience of cooperation between general practice and secondary care when patients are discharged from hospital?	

### Analyses

We used systematic text condensation, a thematic cross-case analysis based on Giorgi, developed and modified by Malterud [29], to analyse the data. It consists of the following steps: 1) reading and listening to all the material and obtaining a total impression; 2) identifying, sorting and coding “meaning units”, units of text providing knowledge of the phenomenon being studied; 3) condensing and abstracting the meaning within each of the codegroups; and, 4) synthesising the condensations into major topics and subtopics that reflect the interviewees’ experiences of causes and consequences of their workload. The main research team consisted of one social scientist, one health economics scientist and four medical doctors, including one academic GP. They participated in all parts of the study, and read and coded the data material separately. Themes, content and coding were discussed thoroughly several times in a plenum, and adjusted by the research team. Academic GPs at our university were involved in the planning process of the study.

### Ethics

No patient information was obtained in this study. The study was approved by the Regional Ethics Committee (2016/2158/REK Midt) and the Norwegian Data Inspectorate (54945). All participants signed a written consent to participate and were given the opportunity to withdraw from the study at any time.

## Results

At the start of this study, our aim was to elucidate the participants’ perceptions of their workload, and the potential explanations and consequences related to variations in workload. However, we noticed that the participants led the discussion into how their perceived workload had increased over the years. They further reflected on the mechanisms for this development. As this was a prominent feature throughout all of the interviews, we chose to let the participants elaborate on this, and integrated it in the further analyses of the material. We categorised the results into three main themes: (1) Heavy and increasing workload – more trend than fluctuation?; (2) Explanations for high workload; (3) Consequences of high workload.

### Heavy and increasing workload – more trend than fluctuation?

Assuming fluctuations in workload, we asked the participants to identify what characterised periods of heavy workload. The participants all described variations in workload, both over weekdays and seasons. Both groups listed epidemics like influenza, with a higher inflow of patients, as resulting in increased workload. Particularly busy periods often occurred for GPs when they or their colleagues had a leave of absence or were preparing for or returning from one.

*“It’s almost like you can’t be away for more than two days, because when you return, the pile of things to do almost feels impossible to handle.”* I, female 1, GP

The co-workers, on the other hand, experienced higher workload when all the doctors at the office were present and thus there was a high turnover of patients. Furthermore, unplanned absence among the doctors was listed as a source of stress and increased workload for the co-workers, because they could not offer any appointments to the patients. The GPs reported now having longer working hours than before, and this despite many of them having reduced the number of patients on their lists. The participants were all experiencing heavy workload at the time of their interviews. GPs from both the focus groups and the individual interviews reported their current situation to be unsustainable.

*“I think things can’t go on like this. I have reached a threshold of what I can fulfil; I think something drastic has to change. […] You get so tired, because you’re half an hour late all the time. It’s like a ‘rat race’ really.”* I, female 9, GP

However, GPs from two of the focus groups experienced their current workload as sustainable, despite increasing. They reflected upon this sustainability as being associated with the way they were organised. One of these practices was managed by the municipality, and the other had recently been reorganised, leaving the managerial position to a medical secretary. The GPs suggested that this allowed them more time for patient contact, as they were relieved from handling some of the administrative tasks such as financial matters, and sick leave among their co-workers.

### Explanations for high workload

The participants reflected upon many possible explanations for the high and increasing workload. Notably, the contributory factors suggested as being most important varied among GPs within the same focus groups and when interviewed individually. The GPs often pointed to “local challenges”, such as having many patients with complex issues, collaborating with the local hospital, and handling administrative and management duties. However, they hardly ever referred to how colleagues with similar challenges had handled these. The co-workers supported the GPs’ explanations, but they also shared more general views on how they perceived societal developments as affecting their working conditions. Below, we give an overview of the mechanisms suggested as creating higher workload, divided into three prominent themes.

#### Transfer of tasks

The participants experienced increasing transfer of medical tasks from secondary to primary care. Follow-ups for patients with cancer and chronic conditions were generally perceived as meaningful, but also challenging and time-consuming. Many participants expressed vexation towards the transferral of more administrative tasks such as writing sick-leave certificates or transport requisitions related to their patients’ hospital visits. The GPs experienced an increasing demand for new diagnostic investigations and tests, both prior to referral and after treatment in secondary care. They gave examples of discharge reports from secondary care instructing the GP to refer the patient to another specialist or radiological examination, thus causing extra workload. This was often perceived as a consequence of a more fragmented and subspecialised secondary care, focusing on shortening hospital stays, and it contributed to a feeling of impaired autonomy. Some GPs stated that they sometimes felt like they were working in “both primary and tertiary care”, being expected to help patients with problems that could not be solved in secondary care.

*“For instance, if we send a patient because of a stomach-ache, they do a gastroscopy, and if they don’t find anything, they send him back, instead of taking care of the problem, like ‘can it be something else?’, and try to find out themselves, like they used to do before. Now they always bounce the ball back in our corner, and we have to do everything ourselves anyway!”* I, female 10, GP

Nevertheless, the GPs acknowledged that secondary-care professionals also have a high workload and do not necessarily intend to be condescending. Communication between primary and secondary care was commonly identified as challenging and time-consuming. This was a well-known problem, but was now perceived to have a higher impact on the workload, as the time pressure was higher. Difficulties in reaching and conferring with secondary-care professionals were thought to result in potentially unnecessary referrals.

*“So I think many referrals could have been avoided, if they had time, and you didn’t have to spend time in line on the phone.”* G8, female 23, GP

#### Increased work per patient

The participants experienced an increasing amount of work per patient in recent years. Changes in legislation, developments in medicine, increasing investigation and treatment possibilities, a need for communication and cooperation with other parts of the healthcare system, and higher demand for documentation were all perceived as contributing factors.

*“Something that has changed in very few years is that there is a lot more work to each patient. (…) Now there is a lot more we can do, (…), and then we had the Coordination Reform, with clearer commands in the discharge reports.”* G8, female 21, GP

While some of the new tasks were regarded as important for patient care, others were perceived as meaningless and bureaucratic. An example frequently mentioned by GPs was writing health certificates.

*“I don’t need a medical degree to document that someone had a cough three days ago (...) nor to write a health certificate for parking needs for someone who has no legs (…) as doctors we have to do something reasonable.”* G2, male 5, GP

The sum of these statutory tasks and demands was seen as a threat to the GPs’ autonomy. The co-workers also reported that they were “writing and writing and writing” to document the work of their practice, although they did not believe this would improve patient health. There seemed to be a general consensus that administrative tasks and “paperwork” had increased considerably over the last decade:

*“The workload comes mostly from the paperwork. I sit with paperwork until seven or eight o’clock every evening. I’m done with patients about four o’clock, so it’s the paperwork that makes it impossible to pick up the kids, or cook dinner…”* I, female 1, GP

#### Changes in society

The participants reflected upon societal changes as explanations for the increasing workload. In general, both the GPs and co-workers experienced increasing patient expectations for healthcare services, treatment options, and their general health and well-being. The co-workers suggested that a lack of family support and limited social networks often resulted in an increased number of doctor visits. They gave examples of minor issues that previously could be solved by “asking grandmother”.

*“People see their GP much more often nowadays. (…) Now you see the doctor at once – if you’ve been feeling ill for a few days (…) If a child gets a rash, then the parents go straight to the doctor to check it out. They didn’t do that before. Now they demand an answer – ‘What is this?’”* I, female 10, GP

Some of the younger GPs suggested that the feeling of time pressure throughout the day resulted in many GPs preferring not to work as many hours as they had previously, similar to others in the society. On the other hand, some of the more experienced co-workers thought the doctors worked even more now and had higher competence in meeting patients’ expectations.

*“Today’s GPs are different to those of 20 years ago. Before, they were mostly elderly, and men. Now, there are many women, and many young people with kids and completely different priorities. They want to go home at a decent time, pick up the kids, make dinner and drive to football practice.”* G2, male 2, GP

The GPs reported that they experienced administrative and economic duties in the GPs’ offices to have become more advanced and complicated in the latest years. They perceived it as more demanding to handle employer responsibilities, such as dealing with pensions and sick leave for their staff. In addition, the expenses for running their offices had increased in recent years due to, e.g., increased requirements for electronic equipment and salaries for employees. As one experienced GP said:

*“It has changed totally. And the capitation fee covers less and less of our real expenses. (…) I used to do my own accounting, but now I can’t possibly do it, because so much has changed. It’s more like running a company. That’s not what I intended to do (laughs). So considering this, it was much easier to be publicly employed.”* I, female 10, GP

This caused economic worries for the GPs, and prevented them from reducing their patient lists and, hence, their workload, because parts of their financing are based on the size of their patient lists.

### Consequences of high workload

Both the GPs and co-workers expressed that they now perceived busy days as the “new normal”. As a response to this, the GPs said they were forced to adjust their way of working by prioritising harder. They prioritised patient consultations, postponed documentation and administrative work to evenings and weekends, and were left with little time for personal rest and recuperation. System-level work, such as participating in meetings, forums and other arrangements at the municipal level, and preventive care were given less priority due to lack of time. Further, the GPs expressed worries about their professional development being negatively impacted through, for instance, postponing or skipping educational courses.

#### Consequences for patients and the healthcare system

Both GPs and co-workers described how high workload had general consequences for patients, such as longer waiting times for appointments, reduced continuity of care due to use of locums, and possibly reduced patient satisfaction. They also shared their thoughts regarding how high workload could lead to suboptimal handling of some patient groups, such as patients with chronic illnesses or complex problems, the elderly, patients with mental health problems and patients with a minority background.

*“It does affect the patients, definitely – regarding waiting times, availability, phone calls and, to some extent, the treatment and the care they receive.”* G2, male 3, GP

We found three different perspectives among the GPs regarding whether and how heavy workload influenced their own clinical decision-making, such as diagnostics, referrals, prescriptions and sick leave. All three perspectives were generally represented by different individuals within the focus groups. There did not seem to be any consistency regarding how these perspectives were related to GPs’ characteristics such as age, experience, gender, or geographical location.

The first perspective was that heavy workload definitely influenced clinical decisions. The GPs gave examples of a lower threshold for prescribing antibiotics to children, and for referring patients with conditions that could have been treated in general practice, such as excessive ear wax and potential deep vein thrombosis. As a female GP said:

*“We try not to do it. We are all quite experienced here, but it’s hard to resist when there is so much to do.”* G7, female 14, GP

The GPs reflected upon how this could have paradoxical effects, and in turn cause more work for the healthcare system and themselves. They were conscious of their gatekeeper function, and described increased referrals as an unfortunate trend they wished to avoid.

*“The more time you have, and the better you’re feeling, both in private and at work, the more guts you have to keep calm and unaffected, which is the art of general practice. And then it’s the gatekeeper function – we have to make sure we don’t refer too many patients – both for the sake of the patients and for the community.”* G8, female 21, GP

The second perspective we found was that heavy workload partially influenced clinical decisions. These GPs were worried that time pressure affected how they interacted with the patients and increased their tendency to take resource-demanding shortcuts in medical investigations. They proposed that stress throughout the work day could increase the risk of making mistakes, or prioritising incorrectly. However, they did not believe that decisions such as referring patients were affected.

*“It’s about being present in the consultation. When in a hurry, you keep more distanced. Maybe you try to find some shortcuts to get things done in a shorter time.”* G10, female 31, GP

The third perspective among the GPs was that heavy workload did not influence clinical decision-making at all, and that the patients were not affected directly.

*“We have to state that the patient is our first priority, and that’s why our days look like they do.”* G2, male 4, GP

All GPs expressed their belief that the trend of heavy and increasing workload had negative impacts on the healthcare system, especially through recruitment problems in general practice.

*“I think it is a symptom that we can’t recruit enough GPs, and then there will be a huge problem in some years.” G2, male 5, GP*

#### Personal consequences for GPs

GPs with children expressed problems regarding combining their job with family life. Many experienced difficulties in getting to kindergarten or school before closing time, finding time to eat dinner with their family, and taking part in their children’s recreational activities. These GPs underlined the importance of having a partner with flexible working hours, so that they could stay at the office for as long as required. GPs without family responsibilities said they felt this was an advantage when the workload was high, and that they could relieve their colleagues when needed.

The GPs described that the workload had consequences for their own health and well-being. At work, they often skipped coffee breaks, shortened their lunch break, and postponed toilet visits. At home, some said that they felt exhausted, easily irritated and stressed, and did not find time to exercise. Two of the younger female GPs worried about being burned out, and not being able to continue working as a GP in the future.

*“Maybe I can stay another year or so, because I love my job. […] I just need some space to breathe in my working day; otherwise I think I will burn out.”* I, female 9, GP

Similarly, the co-workers also felt stressed at work when the workload was high. However, in contrast to the GPs, they also highlighted how they did not have to bring this stress home with them, and they spoke positively of their regulated work hours. The GPs described their decreased motivation for continuing with their job, and a young male doctor said that, based on his experiences of the last year, he no longer wanted to be a GP. Several GPs had considered quitting or were looking for other jobs. Nevertheless, all of our participating GPs expressed a genuine love for their work, felt that their job was meaningful, and wished that conditions would improve so that they could continue.

*“Yes, it’s a wonderful job where you meet all these incredibly nice people that you wouldn’t have met otherwise. It is varied, gives lots of challenges, both in medical and organisational terms. (…) In many ways, it is the best job in the world – you even have an illusion of autonomy (laughs).”* G10, male 33, GP

## Discussion

### Key findings

Our main finding was that the participants perceived the workload in general practice as heavy and having substantially increased in recent years. The suggested explanations for and consequences of heavy workload seemed to vary among the GPs, but they all experienced an increased workload per patient. The participants expressed concerns for the future in regards to patient safety, GPs’ health and motivation, and the recruitment of new GPs.

### Strengths and limitations

A strength of this qualitative approach was that our research group consisted of researchers both with and without clinical experience from general practice. We believe that this balanced our preconceptions, subjective views, and experiences related to clinical practice. It may have also enabled us to recognise various aspects of the topic and potentially led to a more thorough understanding of both the research question and the material. There was little difference between the opinions expressed in focus groups or in individual interviews. Including the GPs’ co-workers, both in separate focus groups and in a focus group together with GPs, gave us a nuanced view of the topic.

Qualitative studies have known limitations concerning transferability. We included only GPs’ offices in Mid-Norway, as inclusion of participants from a larger geographical area was not feasible within the scope of this study. Among all of the GPs asked to participate, only four declined our request, as they could not find time for the interview.

In the months between planning the study and conducting the interviews, there was substantial media attention regarding workload in general practice in Norway. This might have influenced the way we asked questions during the interviews, as well as the way we interpreted the material. It might also have affected the respondents’ views and thoughts about their working conditions and the workload in their practice, and may possibly have led to a polarisation of the opinions.

### Comparison with existing literature

A clear finding was a perception of heavy and increasing workload. Some of the GPs in our study suggested that this perception could partly be influenced by changes in their mentality and expectations. While being a doctor was previously considered a lifestyle choice, today’s young doctors often see it ‘merely’ as a job [30].A study among doctors working at Norwegian hospitals found this difference in perspectives to be associated with an increased work–life imbalance [31]. Several of the GPs in our study reported a work–life imbalance, particularly those with family responsibilities.

However, our findings of GPs experiencing high and increasing workload are supported by recent statistics. Norwegian GPs’ weekly working hours increased by 7 h from 2014 to 2018, resulting in an average of 56 h. Approximately 50% of GPs report that they are working during weekends, even when off duty [32]. At the national level, the total number of patient consultations in general practice is rising [33]. Simultaneously, the number of patients on GPs’ lists has decreased [23], which can be interpreted as more work per patient.

Similar trends of increasing workload have also been reported in other European countries [34]. In England, several studies have reported increased workload for GPs [14, 15, 35], although one report found a slight reduction in working hours between 2012 and 2015 [36]. In Denmark, both workload and working hours in general practice have increased substantially [37]. In a survey of 25 EU countries, 19 reported that “workload in general practice is unreasonable and unsustainable” [38]. The countries that reported general practice workload as reasonable had a common feature of GPs working 8 h or less per day. In comparison, only 10% of the GPs in Norway have weekly working hours within the Norwegian “norm” of 37.5 [32]. Noticeably, the co-workers in our study spoke positively of their regulated working hours as a counterbalance to the increased stress at work, and there is no known recruitment problem in this profession.

In sum, we found a wide range of possible explanations for the increasing and high workload. This is an important finding, as it implicates the complexity of feasible approaches to relieve the situation. Many of the explanations presented by our participants regarding patient and system factors are described in previous research and reports from other countries [15], and could be seen as part of a more general societal development. Many of the mechanisms are also difficult, if at all possible, to change. One cannot stop the population from ageing, or remove multimorbidity. Changing the public’s expectations of what the health care system can and should help them with is probably needed [39], but this will take time. Although some medical technology is held back for economic and ethical reasons, the trend of more diagnostics and treatment possibilities is not easy to halt or reverse [40]. In our study, the particpants suggested to reduce the number of patients per regular GP in order to reduce the workload. We believe this could relieve the workload, but requires recruitment of a large number of new GPs, which constitutes further public expenditure. On the other hand, if the Regular GP scheme is weakened, this could also potentially result in higher expenditure, as a well-functioning primary health care in general is shown to be crucial for public health at a lower cost for the society [41]. Further, continuity of care have been associated with both lower mortality [42], and lower use of secondary health care services [43].

In response to the increasing workload, the GPs in our study handled the situation by for instance expanding working hours and increasing the number of GPs in their office. They pointed out that most of these changes were only temporarily useful. However, outsourcing the position as daily manager had relieved two of the offices from increasingly administrative and employer duties. We were surprised that the participants seemed to have little knowledge about the different possibilities in for example management forms. We believe sharing experiences like these could be helpful for other GPs.

We found that the GPs had different views on if and to what extent patients and the health care system were affected by heavy workload. It was prominent how they stated that they were willing to go to great lengths to prevent their patients from being directly affected by the heavy workload, and explained this as an important reason for the trend of longer working hours. Previous research has shown that GPs are able to adapt to higher workload during periods of higher demand, like influenza pandemic [44]. However, our participants strongly pointed out that they perceived the current situation with high workload to be a trend more than periodic variation. Exposure to high workload over time, increases the risk of burnout [45, 46], which in turn is shown to be harmful for patient safety [47, 48], and also suggested to be associated with higher referral rates [49]. To our knowledge, there are no recent studies describing the prevalence of burnout among GPs in Norway. Nevertheless, European studies report an increasing number of doctors being burned out [36, 50], and suggests association with increased workload.

Autonomy has earlier been reported as a motivation for choosing a career in general practice [10]. If our finding of impaired autonomy among the GPs is a widespread phenomenon, this may impact the recruitment of GPs. Excessive working hours in general practice have also been suggested to cause lower job satisfaction and give recruitment problems [13]. Taking into account that more than one-third of the GPs in Norway are over the age of 55 [23] along with the recruitment challenges in both rural and urban areas [51], the Norwegian healthcare system and wider society face potential challenges.

Despite their heavy workload, the participants were still enthusiastic about their work and societal responsibilities. Nevertheless, they all had concerns for their future, the Regular GP scheme and, like their colleagues in England [14], the recruitment of new GPs. They perceived the Regular GP scheme’s current situation as unsustainable, and expressed worries that they will not be able to provide the population with the expected level of service for primary care in the future [52], despite the Regular GP scheme previously being regarded as a great success [19–21].

## Conclusions

This study found heavy and increasing workload in general practice in Norway. The explanations appear to be multi-faceted and many are difficult to reverse. The GPs expressed worries that they will not be able to provide the population with the expected care and services in the future.

## Abbreviations

GP: General practitioner
